# Personality traits affecting judgement bias task performance in dogs (*Canis familiaris*)

**DOI:** 10.1038/s41598-018-25224-y

**Published:** 2018-04-27

**Authors:** Shanis Barnard, Deborah L. Wells, Adam D. S. Milligan, Gareth Arnott, Peter G. Hepper

**Affiliations:** 1 Animal Behaviour Centre, School of Psychology, Queen’s University Belfast, Belfast, Ireland; 2 School of Biological Sciences, Queen’s University Belfast, Belfast, Ireland

## Abstract

Certain personality traits (e.g. anxiousness, fearfulness), are known to affect the cognitive processing of environmental stimuli, such as the judgement of ambiguous stimuli (judgement bias). Our aim was to assess if personality traits are predictive of a more or less ‘pessimistic’ or ‘optimistic’ judgement bias in the domestic dog. We assessed dog personality (N = 31) using two validated protocols: the Dog Mentality Assessment (standardised battery test) and the CBARQ (owner-based survey). We used a common task based on the animals’ latency to approach a bowl placed in one of three ambiguous positions (Near Positive, Middle, Near Negative) between a baited (Positive) and a non-baited food bowl (Negative) to assess judgement bias. Linear Mixed Model analyses revealed that dogs scoring higher on sociability, excitability and non-social-fear had shorter response latencies to bowls in an ambiguous location, indicating a more ‘optimistic’ bias. In contrast, dogs scoring higher on separation-related-behaviour and dog-directed-fear/aggression traits were more likely to judge an ambiguous stimulus as leading to a negative outcome, indicating a more ‘pessimistic’ bias. Results, partially consistent with previous findings in humans, indicate that personality plays a role in the cognitive processing of environmental stimuli in the domestic dog.

## Introduction

The extensive literature on human psychology demonstrates how the valence of an individual’s emotional state (affect) appears to influence a number of cognitive functions including attention, memory and judgement^1^. People reporting a negatively valenced affective state (e.g. anxiety) show enhanced attention to threatening stimuli (e.g. images of angry faces), are more likely to retrieve negative memories and to make negative judgements about future events or ambiguous stimuli (‘pessimism’), relative to people in a more positive state^2^. For their subjective nature, affective states are impossible to measure directly in non-human animals. Nevertheless, recent research has used the behavioural, physiological and cognitive changes associated with emotional responses as proxies for their valence^1^. Of interest to this work, is the growing evidence that emotions in animals can influence cognitive functions such as attention and judgement capacities by biasing environment perception^1,3^. In rhesus macaques, for example, it has been observed that shifts in emotional state mediated social attention bias away and towards facial cues of conspecifics^4^.

One of the most widely used paradigms for testing emotional states in non-human animals is the judgement bias task (JBT), based on Harding, Paul and Mendl’s seminal paper on rats^5^. Since then, the JBT has been adapted for a variety of species (including starlings, bees, sheep, pigs and dogs) and in a number of different circumstances^6,7^. The JBT is based on the idea that a subject will show a behaviour indicating anticipation of either relatively positive or negative outcomes in response to affectively ambiguous stimuli^1^. Thus, animals in a negative emotional state would be more likely to interpret the ambiguous cues as predicting a negative event (‘pessimism’) and, vice-versa, animals in a positive emotional state would be more likely to interpret the ambiguous cues as predicting a positive event (‘optimism’)^7^. This decision-making process, i.e. to respond to ambiguous signals, is under the influence of current affective states, which in experimental settings can be controlled and modified by manipulating the environmental conditions (i.e. deprivation to induce fear/anxiety or enrichment to induce pleasure/contentment). For example, rats housed in unpredictable housing conditions, which are known to induce a mild depression-like state, were more pessimistic in judging ambiguous stimuli than those in predictable housing^5^. Similarly, pigs housed in an enriched environment had more optimistic judgement biases, indicative of a more positive affect, than pigs housed in a barren environment^8^. The reduction of fearfulness (induced by drug administration) was also found to reduce pessimistic judgements in lambs^3^.

In addition to affective state, other variables might also influence judgment bias. For example, a large number of studies in humans have shown that there are clear associations between personality traits and emotional experiences^9^. In particular, neuroticism is predictive of negative affect, while extraversion is predictive of positive affect^9^. Personality is likely to be a key element in the decision-making processes of non-human animals. For example, dogs housed in shelter that show indications of heightened separation-related distress (i.e. more anxious) have been found to be more pessimistic than those with fewer indicators of separation-related distress^10^. Other authors have further explored the underlying affective state associated with separation anxiety in pet dogs^11,12^. Karagiannis and colleagues^12^ demonstrated how clinical treatments for separation-related problems did not only improve the behaviour, but also the psychological state, of the patients (i.e. dogs had a more ‘optimistic’ approach to the judgement bias task after the treatment than before the treatment), whereas Müller and colleagues^11^ showed how a short-term manipulation (i.e. owner absence) did not induce a negative cognitive bias in pet dogs suffering from mild separation anxiety. Nevertheless, this link between personality and judgement bias remains largely unexplored in non-human animals. The ability to predict the likelihood of an animal experiencing negative emotions according to its main personality traits could be of high value for applied ethologists and animal welfare scientists alike.

The aim of this study was to investigate the influence of personality traits on the decision-making processes of domestic dogs during a judgement bias task. JBT paradigms have already been used successfully to assess the valence of affective state in dogs^10,12–14^, and there is extensive literature providing standardised protocols for the assessment of their personality traits^15–17^. In this study, we assessed personality using two different approaches: the Dog Mentality Assessment^15^ (DMA), a standardised battery test for direct observations of behavioural traits and the widely used Canine Behavioural Assessment and Research Questionnaire^17^ (CBARQ), an owner-based survey. Both direct assessments and questionnaires have advantages and disadvantages^18^ and may assess a range of different traits according to the purpose they were developed for. In a previous study, the DMA test scores were compared to the CBARQ factors scales, and although some scores were weakly correlated, most (e.g. aggressiveness) were not^19^. Thus, the use of both tools allows the assessment of a more comprehensive range of personality traits.

We predicted that this study would highlight a relationship between dogs’ expectation of a more or less positive outcome and their personality traits, with sociable, playful dogs hypothesised to be more ‘optimistic’, while fearful, anxious dogs more ‘pessimistic’ in their judgements.

## Results

Nine dogs did not reach the learning criterion in the training phase of the JBT, thus the final sample included in the analysis was 31 dogs. Those that succeeded in reaching criterion learned the task in an average of 22 trials (min 15–max 50). The number of training trials needed to successfully learn the task did not correlated with the dogs’ ‘trainability’ factor score from the CBARQ (r = 0.18, p = 0.3). To discard the option that dogs were following odour, the last trial was an empty bowl in the positive location. Wilcoxon ranked test analysis confirmed that dogs were equally motivated to reach the bowl placed in the positive location, even if this was empty (Z = −0.56, p = 0.58). In addition, the side of the room on which the positive cue was presented (i.e. left or right) did not affect the dogs’ running speed towards the bowls in the Positive (P) or Negative (N) positions (P: Z = −1.78, p = 0.08; N: Z = 0.51, p = 0.6).

Using a Linear Mixed Model, we initially analysed whether dogs were differentiating between the five different bowl locations (Positive (P), Near Positive (NP), Middle (M), Near Negative (NN) and Negative (N)) and if individual differences in responding to the test (calculated as the average value between latency to reach the positive and the negative bowl locations for each dog) had an effect on their performance (see Methods for a description of the models).

**Figure 1:**
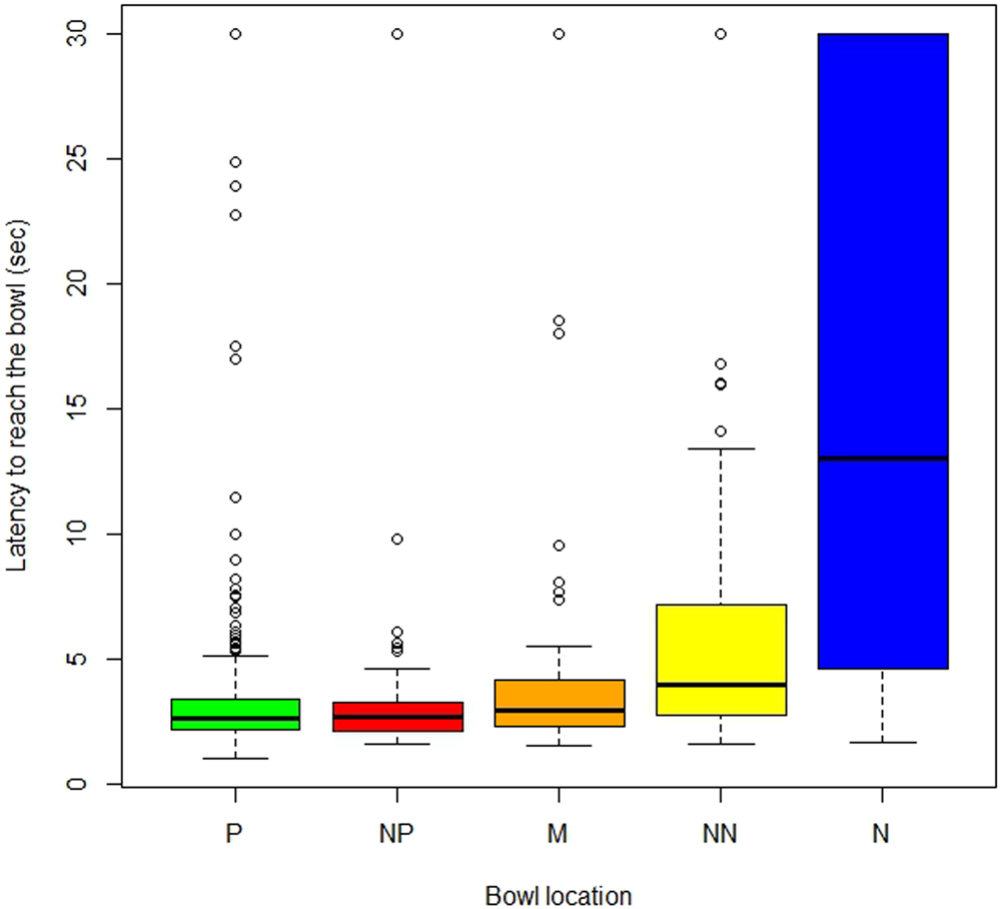
Latency to reach the bowl in the five locations Positive (P) Near Positive (NP), Middle (M), Near Negative (NN) and Negative (N). Results refer to the test trials only. Boxplots represent medians (*bar* within the *box*), upper and lower quartiles (borders of *box*), lowest and highest cases within 1.5 times the IQR (bottom and top *whiskers*) and outliers (*circles*).

Both the bowl position (χ^2^ = 1167.7; p < 0.0001) and the individual differences in performance (χ^2^ = 126.1; p < 0.0001) had a highly significant effect on the dogs’ latency to reach the bowls. Dogs became increasingly slower to reach the bowl the further it was positioned from P (Fig. 1). Post-hoc multiple comparison tests showed that dogs significantly differentiated the time taken to reach the bowl in all locations (p < 0.05) with the exception of the P and NP cues (p = 1.0; see Model 1.1 in Supplementary *Analysis Output* online).

We then checked whether dogs’ characteristics (i.e. sex, age, neutering status and size) influenced the subjects’ performance on the JBT. Model estimates, Wald test for estimates’ p-values and confidence intervals of the fixed effects all confirmed that these traits were not having a significant effect on the outcome variable, i.e. latency (see Model 1.2 in Supplementary *Analysis Output* online).

### Dog Mentality Assessment personality scores and Judgement Bias Task

A first set of models considered the effects of the five personality trait scores from the DMA test (i.e. sociability, playfulness, chase-proneness, curiosity/fearlessness and aggressiveness) on the dogs’ latency to reach the bowl in each of the three ambiguous locations (NP, M, NN). Results showed that there was a significant effect of the sociability trait scores on the animals’ latency to reach the NN probe cues (χ^2^ = 4.68, p = 0.03, 95% CI: low = −0679, upp = −0.016, see Model 2.3 in Supplementary *Analysis Output* online), meaning that dogs scoring higher on this trait were likely to run faster toward the bowl in this location i.e. had a less pessimistic approach. There was no other significant effect of personality trait scores on the dogs’ latency to reach any of the other bowl locations.

### CBARQ factor scores and Judgement Bias Task

In this set of models, we used the same approach as before, this time using the CBARQ factors scores as fixed effects. Results showed a significant effect of dog-directed aggression (DDA) (χ^2^ = 9.34, p = 0.002; CI upp = 0.07, low = 0.40), dog-directed fear (DDF) (χ^2^ = 12.59, p = 0.0004; CI upp = 0.12, low = 0.48), non-social fear (NSF) (χ^2^ = 5.64, p = 0.02; CI upp = −0.62, low = −0.04), separation-related problems (SRP) (χ^2^ = 9.95, p = 0.002; CI upp = 0.12, low = 0.59) and excitability (EXC) (χ^2^ = 11.71, p = 0.0006; CI upp = −0.46, low = −0.11) on the latency to reach the NP cue (see Model 3.1 in Supplementary *Analysis Output* online). A significant effect of EXC (χ^2^ = 4.45, p = 0.03; CI upp = −0.55, low = −0.002) was found on the latency to reach the M cue (see Model 3.2 in Supplementary *Analysis Output* online). Finally, a significant effect of owner-directed aggression (ODA) (χ^2^ = 3.86, p = 0.049; CI upp = −2.10, low = 0.07), DDF (χ^2^ = 4.49, p = 0.03; CI upp = 0.004, low = 0.67) and a tendency of EXC (χ^2^ = 3.68, p = 0.055; CI upp = −0.63, low = 0.03) was found on the latency to reach the NN cue (see Model 3.3 in Supplementary *Analysis Output* online). The more conservative 95% CI approach confirmed all results with the exception of ODA and EXC in the latter set of results (i.e. the upper and lower limits included zero so should not be considered significant). While DDA, DDF, and SRP effect parameter estimates were positive, meaning that an increase in the dogs’ personality scores was associated with an increase in the latency to reach the bowl (i.e. more pessimistic bias with higher severity of the behavioural problem), the EXC, NSF and ODA estimates were negatively associated with latency, meaning that dogs scoring higher on those traits were more likely to run faster (i.e. shorter latency) to the ambiguous cue (model estimates presented in Supplementary *Analysis Output* online).

## Discussion

In this study, we found evidence that there is a relationship between personality traits and cognitive bias in the domestic dog. Our results show that dogs scoring higher on sociability (using the DMA test) and dogs rated as more excitable or prone to non-social-fear by their owners (using the CBARQ) were more likely to display an optimistic judgement of an ambiguous stimulus, while dogs rated higher on separation-related-problems, and dog-directed fear/aggression traits were more likely to show a pessimistic bias. Studies in human psychology have demonstrated that personality traits, and not only emotional states, are associated with the cognitive processing of environmental stimuli^9,20–22^. In fact, in natural mood conditions (i.e. when a person’s mood is not experimentally induced or manipulated), personality dimensions appear to be better predictors of emotional information processing than mood states^20,23,24^.

Our results are partially in line with previous results on humans: people scoring high on ‘positive’ traits (e.g. extraversion, agreeableness) are more likely to make positive judgements^25^, whereas those scoring high on ‘negative’ traits (e.g. neuroticism) are more likely to make negative judgements^24^. The sociability trait scored using the DMA test is characterised by dogs’ willingness to approach and greet unfamiliar people in a friendly way, with higher scores describing exuberant dogs pulling and jumping toward the Experimenter. Together with the ‘excitable’ CBARQ factor, these traits can be compared to the extrovert profile described in humans^26^. In our study, dogs scoring higher on the sociability trait were more likely to run faster to the bowl in the NN location, whereas dogs described as excitable were more likely to make an overall more positive judgement when faced with the three ambiguous cues (i.e. run faster to the bowl in the NP, M and NN (tendency) locations). An opposite result was found for three CBARQ factors (i.e. SRP, DDF and DDA) that can be considered ‘negative’ traits as normally characterised by high levels of anxiety, fear or aggression. Dogs scoring higher on these factors were more likely to judge an ambiguous stimulus in a negative way (i.e. be more pessimistic). Specifically, dogs with higher scores on the DDF factor were increasingly slower in reaching both the NP and NN cue, dogs with higher scores on the SRP factor were increasingly slower in approaching the NP location only and those with higher scores on the DDA factor were increasingly slower in approaching the NN bowl.

Although we made no specific hypothesis on whether different personality types may result in different performances toward specific probe locations, the expectation was that the majority of the difference in running speed would be found towards the bowl in the NN position. This is because the NN cue reflects an indication of high expectation of negative results, normally underlying affective states of fear and anxiety. Alternatively, changes in the speed toward the NP location reflects a low expectation of a reward or positive outcome, normally associated with depression-like states^1^, which was not directly measured by either of the tools we used to assess personality.

Previous works on dogs have investigated the underlying affective state associated with separation-related-behaviours^10–12^. Karagiannis and colleagues^12^ found that the clinical group suffering from SRP ran significantly slower to the NN bowl location. Mendl and colleagues^10^ reported that more anxious dogs ran more slowly to the M bowl location, but not to the NP or NN cues, whereas in our study dogs scoring higher on SRP ran increasingly slower toward the NP cue. Overall, results consistently suggest that higher levels of separation anxiety lead to more pessimistic judgements of ambiguous cues, thus this should be treated as a welfare problem and measures should be taken to relieve the dog from this state. Karagiannis and colleagues^12^, for example, showed how behavioural and pharmaceutical interventions reduced the negative bias toward an ambiguous cue. Nevertheless, there is an evident discrepancy between the studies in the probe cue revealing the pessimistic bias. This could be due to a number of reasons, such as the nature of the study population (i.e. shelter, pets, clinical and non-clinical cases), as well as the study design and location (i.e. shelter, home, laboratory), and/or individual variability. Müller and colleagues^11^ highlighted how there can be a very high individual variability of response to the ambiguous stimuli, with dogs either running progressively slower from NP to M to NN, or running equally fast to two of the probe locations (e.g. NP and M) and then significantly differently to the third (e.g. NN). The individual variability in responding to the probe cues, together with the differences in the type and severity of the behavioural problem may be a cause of inconsistencies in cognitive bias results.

It is important to note that we failed to find significant associations that would have been expected between some of the traits assessed using either tools and the performance on the JBT (e.g. curiosity/fearlessness, playfulness). Also, the association between NSF/ODA and latency to reach the NP cues could be regarded as counterintuitive (we would expect dogs with higher scores on these traits to run slower toward the ambiguous bowls and not faster as the results showed). This could be for a number of reasons, including a motivation to increase the distance from the owner (especially for the ODA dogs) or study limitations which are discussed below.

One explanation for not finding expected associations between personality traits and the JBT response may be in the characteristics of our sample. Although there was a certain amount of variability in the personality trait scores, it is probable that more extreme scores (e.g. extremely aggressive or anxious dogs) were not represented. This is an intrinsic constraint of the experimental design, as the testing of excessively fearful/anxious or aggressive dogs could have compromised the welfare and safety of both dogs and handlers. Future research should explore this link between personality and cognitive bias further, including dogs with more severe behavioural problems. The design could also be adapted to ensure testing is not too stressful, e.g. using attention bias tests instead of judgement bias, which avoids the need for extensive training, and performing tests in the dog’s home environment instead of an unfamiliar testing area.

Although the judgement bias task has proved valid in assessing the long-term mood state of animals, performance may be affected by recent positive or negative experiences which we had not controlled for (e.g. the dog being rewarded or reprimanded by the owner before arriving at the lab). Experiencing an acute stressor, such as restrain, elicited an optimistic judgement in sheep^27^, and a rewarding consumption experience before testing elicited a more pessimistic judgement in dogs^28^. These results are opposite to prediction (see^25,26^ for discussion). Nevertheless, they highlight how even short-term experiences may impact the animal’s perception of an ambiguous cue, thereby leading to contrasting results when comparing JBT studies. Although our animals did not receive any treatment to manipulate their affective state, underlying mood and/or short-lived emotions may well have influenced JBT performance. Working with family pets has the limitation that their immediate quality of live or personal experiences cannot be controlled for, as is the case with laboratory or farm animals.

It appears that none of the ‘negative’ personality traits affected the dogs’ latency to reach the middle bowl. Although this was significantly longer than latency to reach the NP bowl, dogs overall seemed to treat the M bowl as more of a rewarded stimulus (Fig. 1). This could be an artefact of the experimental design, as the Experimenter would stand behind this bowl when the dog was released. Even though the Experimenter was two meters away from the bowl and standing passively, being on the trajectory of the middle bowl might have affected the dogs’ expectation of a reward, or else the mere presence of the person behind the bowl could have attracted the dogs in that direction (even more so if the dogs were friendly and excitable).

In the past decade, the scientific community has shown a lot of attention to measuring emotional processing in non-human animals using cognitive approaches^1,5,6,29,30^. However, so far, the influence that personality may have on cognitive bias tasks has been largely overlooked. Results from the few studies that did include personality in their models, support the findings that stable personality traits do play a role in modulating transient mood states and cognitive outcomes^31,32^. Cussen & Mench^32^, for example, reported that differences in personality dimensions of psittacines (*Amazonica amazonica*) were correlated with differences in cognition, with more neurotic parrots showing a greater attention bias for environmental stimuli. Recently, in their study on pigs (*Sus scrofa domesticus)*, Asher and colleagues^31^ found that mood and personality interact, impacting on judgement. Pigs with a proactive personality were overall optimistic in their judgement of an ambiguous stimulus, independent of housing manipulations designed to influence their mood. Pigs with a reactive personality, however, were significantly affected by their housing conditions, with those in an enriched environment being more optimistic and those in a less enriched environment being more pessimistic than the proactive pigs^31^. This is in line with findings from human studies where high neurotic profiles have stronger affective reactions to unpleasant stimuli, whereas high extravert profiles have stronger positive affective reactions to pleasant stimuli^33^. Manipulating the environment to induce a desired mood state is common practice in both human and non-human animal studies^4,5,8,24^. However, some individuals may be affected more by the manipulation, so future researchers should consider the effect of a possible interaction between mood and personality when assessing cognitive bias.

## Conclusions

This study supports the hypothesis that, as in humans, personality plays an important role in the cognitive processing of environmental stimuli in domestic dogs. It is important that future studies on cognitive bias take into account possible effects of both mood and personality in their statistical models, to increase the reliability of their outcomes.

## Methods

### Ethical Note

All methods adhered to the Association for the Study of Animal Behaviour/Animal Behavior Society Guidelines for the Use of Animals in Research (Association for the Study of Animal Behaviour, 2006). Ethical approval for the study was granted by the Research Ethics Committee, School of Psychology, QUB.

### Animals

Forty privately owned pet dogs were recruited for this study among the students and staff of the School of Psychology, Queen’s University Belfast, and by word of mouth. Dogs comprised 22 males (82% neutered) and 18 females (78% spayed) and included a number of different breeds and breed-crosses. The minimum age of the subjects was 12 months; the oldest dog was 13 years of age (mean age = 4.7 ± SEM 0.47 years).

### Assessment of personality traits

Personality traits were assessed using both a direct (battery test) and indirect method (owner-based survey).

#### Standardised battery test

We followed the methodology described in Barnard *et al*.^34^, which provides an adapted version of the Dog Mentality Assessment (DMA) test^15,35^. The DMA was originally tested on over 15,000 dogs and the factor analysis based on that sample extracted five personality traits: playfulness, curiosity/fearlessness, chase-proneness, sociability and aggressiveness^15^.

During the test, the dog was presented with the following situations: social contact, passive test, play, chase, distance-play, sudden appearance, metallic noise and ghost. The dog’s behavioural reactions were scored according to 32 predefined behavioural variables and each variable was scored from 1 to 5 according to the intensity of the dog’s reaction. The protocol and scoring are described in Supplementary Table S1.

#### Owner based survey

As an indirect method, we used the widely applied Canine Behavioural Assessment and Research Questionnaire^17^ (CBARQ). Owners were asked about their dog’s behavioural responses to specific events or situations in their usual environment. The CBARQ items are 5-point rating scales and were developed to gather information on validated behavioural factors. The factors included in our analysis were: “stranger-directed aggression” (SDA), “owner-directed aggression” (ODA), “stranger-directed fear” (SDF), “dog-directed fear” (DDF), “dog-directed aggression” (DDA), “attachment-attention-seeking” (AAS), “separation-related problems” (SRP), “non-social fear” (NSF), “trainability” (TR), and “excitability” (EXC). The behavioural factors with all the representative CBARQ items and related rating scales are presented in Supplementary Table S2.

### Judgement Bias Task (JBT)

The JBT was performed following Wells *et al*.’s^14^ paradigm.

#### Training

Dogs were initially trained to differentiate the position of a bowl containing a food treat (Positive, P) from the position of an empty bowl (Negative, N). To do so, dogs were introduced to the testing room (5 × 5m) and presented with positive and negative trials in a pseudo-random order. No more than two trials of the same type were presented consecutively. The location of the positive cue was balanced (i.e. 50% on the left and 50% on the right). For each dog, the latency to make contact (i.e., touch or put its head inside) with the bowl was recorded. A trial was terminated once the dog contacted the bowl or 30 seconds had elapsed without the animal approaching the bowl. Each dog received a minimum of 15 training trials and was allowed a maximum of 50 trials to learn the task. Dogs were considered to have learned the task when, for the preceding three positive trials and the preceding three negative trials, the longest latency to reach the positive location was at least half a second shorter than any of the latencies to reach the negative location.

### Testing

**Figure 2:**
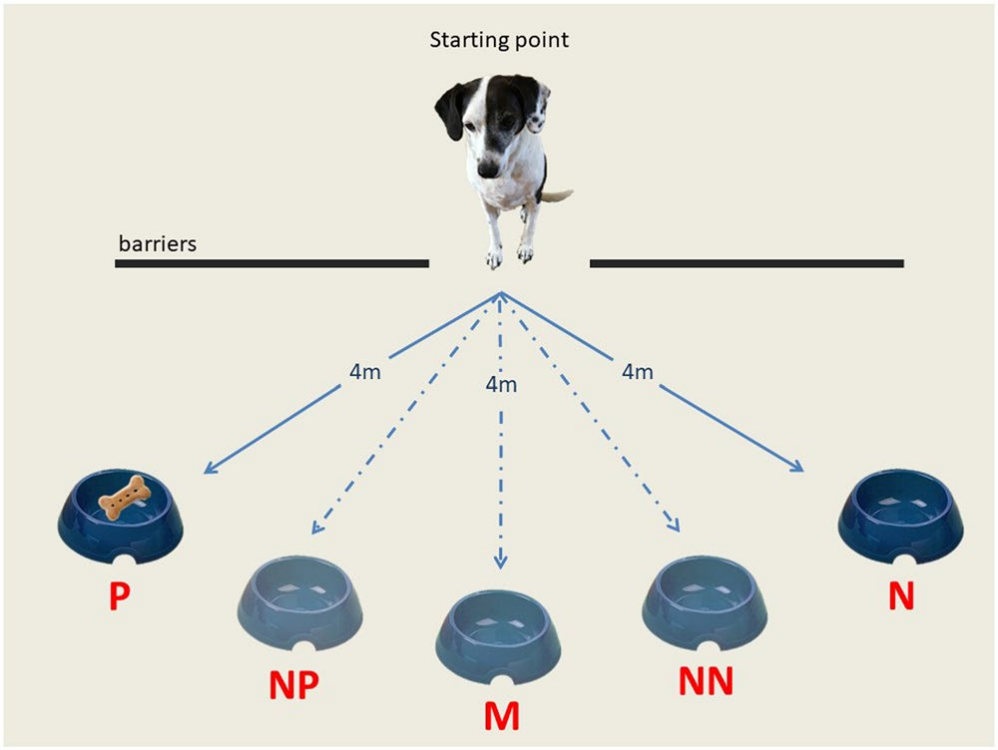
Depiction of the food bowl positions used in the judgement bias task [P = Positive position (bowl baited with food); NP = Near Positive; M = Middle; NN = Near Negative; N = Negative position (bowl devoid of food)].

The testing phase commenced immediately after successful training to criterion. Dogs were presented with a food bowl (empty) placed in one of three ambiguous locations between the original Positive (P) and Negative (N) cues: Near Positive (NP), Middle (M) and Near Negative (NN). All probe locations were presented three times, separated by 4 standard training trials (i.e. P, N) (Fig. 2). We were interested in analysing the dogs’ response to all three probe locations, as previous literature suggested that differences in the performance toward specific probe locations may underlie different affective states^1,36^.

### Analyses

Following the results from Svartberg and Forkman^15^ and our previous work^34^, we calculated the dogs’ trait scores for the following personality traits: playfulness, curiosity/fearlessness, chase-proneness, sociability and aggressiveness. The dog’s score (1–5) on each variable was standardized using *z*-scores^35^. Then, the standardized values for the representative variables of each factor (i.e. variables with high loadings on a factor, according to the results in Svartberg and Forkman^15^) were averaged to calculate dogs’ personality trait scores. Table S1 shows which are the representative variables for each personality trait.

A private account on the official online platform (cbarq.edu) was used to collect the CBARQ data and the output scores were automatically generated by the web site. These output scores are calculated as the average of the questionnaire items loading highly on each factor as described in Hsu & Serpell^17^ and were used untransformed in the analyses.

To discard any methodological bias, we used a Wilcoxon ranked test for related samples to check that dogs were not following odour cues, i.e. comparing the latencies to reach the bowl in P when it was either rewarded or un-rewarded (i.e. last test trial presented with an empty bowl in P). A Mann-Whitney U-test was used to check that the latency to reach the bowl in P and N was not affected by the side of the room in which these cues were presented. Additionally, we assessed whether training success in the JBT (i.e. the number of trials needed to reach criteria) was significantly correlated (using Spearman rank test) to the dogs’ trainability (assessed using the CBARQ ‘trainability’ factor score).

Before investigating any effect of personality on the performance of dogs on the JBT, we used linear mixed-effect models to: (a) confirm that dogs were differentiating between the 5 different cues, i.e. that latency was significantly affected by the bowl location; and (b) investigate if performance on the JBT was affected by specific dogs’ characteristics, i.e. sex, dog size, neutering status.

For the first model, latency to reach the bowl was the outcome variable, using log-transformation to normalise model residuals. To control for individual differences in running speed (i.e. due to size or motivation) we calculated the average value between the latency to reach the positive and the negative bowl locations for each dog and used this value as a covariate fixed factor (consistent with^36^); dog identity was added as random factor. Maximum-likelihood estimation was used to account for imbalanced data^6^ (16 trials for the P and N cue location and 3 trials for each probe location).

To check if there was any effect of dog characteristics on performance, a second model was created with again log-transformed latency data as the outcome variable, and bowl location, dog sex, age, size and neutering status as fixed effects. Because, as expected, running speed had a highly significant effect in our first model, this value (mean latency between P and N) was included in all subsequent models as a random factor in addition to dog identity.

Given the nature of our hypothesis, to assess whether different personality traits would affect the latency to reach the bowls placed in the ambiguous locations (NP, M, NN) and simplify the models given our sample size, we stratified the data-set and analysed each bowl position separately. Also, as the methods used to assess dogs’ personality were very different (i.e. a battery test and an owner-based survey), we created two separate analyses, the first set of models included the personality variables derived from the DMA test, and the second set of models included the factor scores derived from the CBARQ. This way, each model would look at the effect of the personality traits on the latency to reach the bowl in one of the three probe locations, avoiding the need to add personality-bowl location interaction effects. Again, latency was the outcome variable and mean latency between P and N and dog identity were added as random factors with maximum-likelihood estimation. P-values for the fixed effects were extracted by a Wald Chi-square test (‘car’ package) and fixed-effect model estimates were further discussed by checking their 95% confidence intervals (CI). Where appropriate, post-hoc analyses (‘multcomp’ package) were used for multiple pair comparisons.

R 3.4.3 (R Development Core Team) was used for analysis, with *alpha* = 0.05.

### Data Availability Statement

The datasets analysed during the current study are available from the corresponding author on reasonable request.

## Electronic supplementary material

Supplementary Information
