# Supplementary material for: Personality traits affecting judgement bias task performance in dogs (Canis familiaris)
Source: Sci Rep. 2018 Apr 27;8:6660. doi: 10.1038/s41598-018-25224-y (PMC5924375; doi:10.1038/s41598-018-25224-y)
Supplement: Supplementary file 1 — Supplementary Information [file 41598_2018_25224_MOESM1_ESM.pdf]

Personality traits affecting judgement bias task performance in dogs (*Canis familiaris*)

Shanis Barnard, Deborah L. Wells, Adam DS Milligan, Gareth Arnott and Peter G. Hepper

Table S1. Dog Mentality Assessment test. Outline of subtests, variable scores and personality traits they refer to (adapted from Svartberg & Forkman 2002. *Appl Anim Behav Sci 79, 133-155*).

Subtest description	Behavioural variables (#)	Personality trait
**Social contact:** The dog and owner approached the TL, who greeted the owner and the dog. The TL took the leashed dog for a short walk, during which she stopped and petted the dog. Back with the owner, the TL made a brief handling of the dog.	(1) Greeting reaction<br>(2) Following behaviour<br>(3) Physical handling	Sociability<br>Sociability<br>Sociability
**Passive test:** The owner and the TL sat on chairs at the opposite side of a room. The owner unleashed the dog, which was free to explore the area for 3 min. The owner and TL remained passive for the whole time.	(4) Activity	-
**Play 1**: The dog was unleashed in a room, and a rag was thrown between the owner and the TL, and then away from the dog. If the dog ran after and caught the rag, the TL tried to call the dog back. This procedure was repeated once. After the repetition, the dog was invited to play tug-of-war with the TL.	(5) Interest in play<br>(6) Intensity of grabbing<br>(7) Interest in laying tug-of-war	Playfulness<br>Playfulness<br>Playfulness
**Chase:** A prey-like puppet was fixed to the loose end of a long flexi leash (~8m), which was operated by the RA from a hidden location. Owner and dog approached the starting-point, when the dog spotted the prey, the owner released the dog and the RA unlocked the flexi leash so that it winded dragging the prey. The dog was free to run after it. The object stopped when the leash was fully winded, the RA left the pray on the floor for the dog to grab. The test was repeated once.	(8) Interest in following 1<br>(9) Grabbing behaviour 1<br>(10) Interest in following 2<br>(11) Grabbing behaviour 2	Chase-proneness<br>Chase-proneness<br>Chase-proneness<br>Chase-proneness
**Distance-play**: The RA dressed in a cape with a hood, moved and crouched 3 times about 10 m from the owner and the leashed dog. Then he unhooded and tossed a rag in the air, turned, ran a short distance to a hiding place and called the dog. The dog was then unleashed so that it was free to approach the RA. If this happened, the assistant played tug-of-war with the dog using the rag, then was passive for 10 s. The play and passivity phase was repeated once.	(12) Interest in the person<br>(13) Aggressive behaviour<br>(14) Exploratory behaviour<br>(15) Attempts to play tug-of-war<br>(16) Play invitations	-<br>Aggressiveness<br>-<br>-<br>-

**Sudden appearance**: During a walk by the owner and leashed dog, a humanlike dummy was suddenly dropped from the ceiling down in front of the dog at a distance of 2 m. When the dummy dropped, the owner was instructed to release the grip of the leash. The dog was free to escape from or explore the dummy. If the dog did not approach the dummy by itself, the owner supported the dog in four successive steps (Svartberg & Forkman, 2002) or until the dog had investigated the dummy. Thereafter, handler and dog walked close to the dummy four times.	(17) Startle reaction<br>(18) Aggressive behaviour<br>(19) Exploratory behaviour<br>(20) Avoidance behaviour<br>(21) Approach behaviour	Curiosity/fearlessness<br>Aggressiveness<br>Curiosity/fearlessness<br>Curiosity/fearlessness<br>-
**Metallic noise:** During a walk by the owner and leashed dog, a chain with large links was dragged over a sheet of corrugated metal 2 m from the dog. Thereafter, a similar familiarisation procedure as in the subtest Sudden Appearance was carried out.	(22) Startle reaction<br>(23) Exploratory behaviour<br>(24) Avoidance behaviour<br>(25) Approach behaviour	Curiosity/fearlessness<br>Curiosity/fearlessness<br>Curiosity/fearlessness<br>-
**Ghost:** The RA wearing white sheet and with a white plastic hockey mask over his head moved slowly towards the leashed dog and the owner. The distance between the RA and the dog was 10 m at the beginning of the test. The ghost moved in short, intermittent stages towards the dog until he was 2 m close. Then, the dog was released and could freely investigate the ghost, who removed the sheet and mask when the dog approached.	(26) Attention towards ghost<br>(27) Aggressive behaviour<br>(28) Avoidance behaviour<br>(29) Exploratory behaviour<br>(30) Greeting behaviour	Aggressiveness<br>Aggressiveness<br>Curiosity/fearlessness<br>-<br>Sociability
**Play 2**: This subtest was a repetition of the subtest Play 1, except that the tug-of-war was eliminated	(31) Interest in play<br>(32) Intensity of grabbing	Playfulness<br>Playfulness

TL=test leader; RA=research assistant

[-] indicates that variable did not load on any personality trait in the original factor analysis (Svartberg & Forkman 2002)

Table S2. CBARQ. Description of the items grouped in the 10 factor scores categories used in the analysis

1. Trainability (TR)

Dog
- Returns immediately when called while off leash
- Obeys a sit command immediately
- Obeys a stay command immediately
- Will fetch or attempt to fetch sticks, balls, and other objects
- Seems to attend to or listen closely to everything the owner say or does
- Is slow to respond to correction or punishment
- Is slow to learn new tricks or tasks
- Is easily distracted by interesting sights, sounds, or smells

2. Stranger-Directed Aggression (SDA)

Dog acts aggressively
- When approached directly by an unfamiliar adult while being walked or exercised on a leash.
- When approached directly by an unfamiliar child while being walked or exercised on a leash
- Toward unfamiliar persons approaching the dog while it is in the owner's car
- When an unfamiliar person approaches the owner or a member of the owner's family at home
- When an unfamiliar person approaches the owner or a member of the owner's family away from home
- When mailmen or other delivery workers approach the home
- When strangers walk past the home while the dog is in the yard
- When joggers, cyclists, roller skaters, or skateboarders pass the home while the dog is in the yard
- Toward unfamiliar persons visiting the home
- When unfamiliar person tries to touch or pet the dog

3. Stranger-directed fear (SDF)

Dog acts anxious or fearful
- When approached directly by an unfamiliar adult while away from the home
- When approached directly by an unfamiliar child while away from the home
- When unfamiliar person tries to touch or pet the dog
- When unfamiliar persons visit the home

4. Owner-directed aggression

Dog acts aggressively
- When verbally corrected or punished by a member of the household
- When toys, bones, or other objects are taken away by a member of the household
- When bathed or groomed by a member of the household
- When approached directly by a member of the household while it is eating
- When food is taken away by a member of the household
- When stared at directly by a member of the household
- When stepped over by a member of the household
- When a member of the household retrieves food or objects stolen by the dog

5. Dog-directed aggression (DDA)

Dog acts aggressively
- When approached directly by an unfamiliar male dog while being walked or exercised on a leash
- When approached directly by an unfamiliar female dog while being walked or exercised on a leash
- Toward unfamiliar dogs visiting the home
- When barked, growled or lunged at by an unfamiliar dog

6. Dog-directed fear (DDF)

Dog acts anxious or fearful
- When approached directly by an unfamiliar dog of the same or larger size
- When approached directly by an unfamiliar dog of a smaller size
- When unfamiliar dog visit the house

When barked, growled or lunged at by an unfamiliar dog

7. Nonsocial fear (NSF)

Dog acts anxious or fearful

In response to sudden or loud noise
In heavy traffic
In response to strange or unfamiliar objects on or near the sidewalk
During thunderstorms
When first exposed to unfamiliar situations
In response to wind or wind-blown objects

8. Attachment-attention-seeking (AAS)

Dog

Displays a strong attachment for a particular member of the household
Tends to follow a member of household from room to room about the house
Tends to sit close to or in contact with a member of the household when that individual is sitting down
Tends to nudge, nuzzle, or paw a member of the household for attention when that individual is sitting down
Becomes agitated when a member of the household shows affection for another person
Becomes agitated when a member of the household shows affection for another dog or animal

9. Separation related problems (SRP)

Dog displays

Shaking, shivering, or trembling when left or about to be left on its own
Excessive salivation when left or about to be left on its own
Restlessness, agitation, or pacing when left or about to be left on its own
Whining when left or about to be left on its own
Barking when left or about to be left on its own
Howling when left or about to be left on its own
Chewing or scratching at doors, floor, windows, and curtains when left or about to be left on its own
Loss of appetite when left or about to be left on its own

10. Excitability (EX)

Dog overreacts or is excitable

When a member of the household returns home after a brief absence
When playing with a member of the household
When the doorbell rings
Just before being taken for a walk
Just before being taken on a car trip
When visitors arrive at its home

Behavioural categories 2-7, 9 and 10 were scored on five-point qualitative scales: 0= no sign of the asked behaviour, 1 to 3= mild to moderate sign of the behaviour, 4= high/severe sign of the behaviour.

Behavioural categories 1, 8 and 10 were scored on a rating scale including five options, grading ‘‘never’’, ‘‘seldom’’, ‘‘sometimes’’, ‘‘usually’’ and ‘‘always’’.

Analysis Output (R 3.4.3)

NOTE: these are not the full analyses but just the relevant outputs mentioned in the main paper. Please refer to the paper for detail description of the statistical analyses.

Model 1.1

```
#We assessed if dogs were able to differentiate between the different bowl locations (var
 name=Index1). Mean latency between positive and negative location (var name= MEAN) was added as
 covariate to control for differences in running speed.#

> model1<-lme(log_latency~Index1+MEAN,,data=data,random=~1|Dog,method="ML")
> summary(model1)
Linear mixed-effects model fit by maximum likelihood
 Data: data
       AIC      BIC    logLik
  2648.008 2689.189 -1316.004

Random effects:
 Formula: ~1 | Dog
        (Intercept)  Residual
StdDev:   0.1082378 0.6755014

Fixed effects: log_latency ~ Index1 + MEAN
                 Value  Std.Error   DF   t-value p-value
(Intercept)  0.4653468 0.10652981 1235  4.368231  0.0000
Index1N      1.1092269 0.07651192 1235 14.497438  0.0000
Index1NN     0.3504342 0.09929498 1235  3.529224  0.0004
Index1NP    -0.2841815 0.09929498 1235 -2.861993  0.0043
Index1P     -0.2830809 0.07651192 1235 -3.699828  0.0002
MEAN         0.0857170 0.00765041 1235 11.204234  0.0000
 Correlation:
         (Intr) Indx1N Ind1NN Ind1NP Indx1P
Index1N  -0.605
Index1NN -0.466  0.649
Index1NP -0.466  0.649  0.500
Index1P  -0.605  0.842  0.649  0.649
MEAN     -0.729  0.000  0.000  0.000  0.000

Standardized Within-Group Residuals:
        Min          Q1         Med          Q3         Max
-2.99685958 -0.63147436 -0.07544622  0.64522073  3.62655423

Number of Observations: 1271
Number of Groups: 31

#We run an ANOVA on the linear part of the model [library(car)] to calculate p-values for the fixed effects
 using Wald test#

> Anova(model1)
Analysis of Deviance Table (Type II tests)

Response: log_latency
         Chisq Df Pr(>Chisq)
Index1 1167.73  4  < 2.2e-16 ***
MEAN    126.13  1  < 2.2e-16 ***
---
Signif. codes:  0 '***' 0.001 '**' 0.01 '*' 0.05 '.' 0.1 ' ' 1


#We run a Post-hoc test for multiple comparisons to assess if dogs were differentiating between the bowl
 positions#

> model1PH<-glht(model1,linfct=mcp(Index1="Tukey"))
> summary(model1PH)

  Simultaneous Tests for General Linear Hypotheses

Multiple Comparisons of Means: Tukey Contrasts

Fit: lme.formula(fixed = log_latency ~ Index1 + MEAN, data = data,
    random = ~1 | Dog, method = "ML")

Linear Hypotheses:
              Estimate Std. Error z value Pr(>|z|)
N - M == 0     1.109227   0.076331  14.532  < 0.001 ***
NN - M == 0    0.350434   0.099060   3.538  0.00339 **
NP - M == 0   -0.284182   0.099060  -2.869  0.03056 *
P - M == 0    -0.283081   0.076331  -3.709  0.00174 **
NN - N == 0   -0.758793   0.076331  -9.941  < 0.001 ***
NP - N == 0   -1.393408   0.076331 -18.255  < 0.001 ***
P - N == 0    -1.392308   0.042894 -32.459  < 0.001 ***
NP - NN == 0  -0.634616   0.099060  -6.406  < 0.001 ***
P - NN == 0   -0.633515   0.076331  -8.300  < 0.001 ***
P - NP == 0    0.001101   0.076331   0.014  1.00000
---
```

```
Signif. codes:  0 '***' 0.001 '**' 0.01 '*' 0.05 '.' 0.1 ' ' 1
(Adjusted p values reported -- single-step method)
```

Model 1.2

```
#We checked If dogs' characteristics (i.e. sex, age, neutering status and size) had an effect on
 the latency to reach the bowls#

> model2<-
 lme(log_latency~Index1+DogSize+NeutSpay+Sex+Age,,data=data,random=~1|Dog/MEAN,method="ML",na.action=na.exclude)
> summary(model2)
Linear mixed-effects model fit by maximum likelihood
 Data: data
       AIC      BIC    logLik
  2701.845 2768.764 -1337.923

Random effects:
 Formula: ~1 | Dog
        (Intercept)
StdDev:   0.1196209

 Formula: ~1 | MEAN %in% Dog
        (Intercept)  Residual
StdDev:   0.2702476 0.6738842

Fixed effects: log_latency ~ Index1 + DogSize + NeutSpay + Sex + Age
                 Value  Std.Error   DF   t-value p-value
(Intercept)  1.3885805 0.17316160 1206  8.018986  0.0000
Index1N      1.0829985 0.07912890 1206 13.686510  0.0000
Index1NN     0.3242058 0.10129310 1206  3.200671  0.0014
Index1NP    -0.3104099 0.10129310 1206 -3.064472  0.0022
Index1P     -0.3093093 0.07912890 1206 -3.908929  0.0001
DogSize2    -0.2246766 0.17156490   25 -1.309572  0.2022
DogSize3    -0.2038878 0.12349146   25 -1.651027  0.1112
NeutSpay2    0.0457153 0.15700282   25  0.291175  0.7733
Sex2        -0.0475524 0.11142798   25 -0.426754  0.6732
Age          0.0202203 0.02216905   25  0.912097  0.3704

Standardized Within-Group Residuals:
        Min          Q1         Med          Q3         Max
-2.94391713 -0.58340113 -0.09445352  0.56133564  3.67398411

Number of Observations: 1271
Number of Groups:
          Dog MEAN %in% Dog
           31            61

#We run an ANOVA on the linear part of the model [library(car)] to calculate p-values for the fixed effects
 using Wald test#
> Anova(model2)
Analysis of Deviance Table (Type II tests)

Response: log_latency
             Chisq Df Pr(>Chisq)
Index1    1168.4800  4     <2e-16 ***
DogSize      3.1770  2     0.2042
NeutSpay     0.0855  1     0.7700
Sex          0.1836  1     0.6683
Age          0.8385  1     0.3598
---
Signif. codes:  0 '***' 0.001 '**' 0.01 '*' 0.05 '.' 0.1 ' ' 1

#We investigated if the model would improve by using a backward stepwise approach (i.e. dropping variables one
at the time and comparing the AIC values for best fit). The model fit did not improve significantly so we used
the above model as the final one#

#To further explore the reliability of our results we compared them with the confidence intervals of the model
estimates [significant effect is considered if the CI does not include zero]#
> intervals(model2,which="fixed")
Approximate 95% confidence intervals

 Fixed effects:
                  lower        est.       upper
(Intercept)  1.05018810  1.38858045  1.72697281
Index1N      0.92836484  1.08299852  1.23763220
Index1NN     0.12625891  0.32420585  0.52215278
Index1NP    -0.50835683 -0.31040990 -0.11246296
Index1P     -0.46394294 -0.30930927 -0.15467559
DogSize2    -0.57662833 -0.22467657  0.12727520
DogSize3    -0.45722067 -0.20388775  0.04944517
NeutSpay2   -0.27636350  0.04571531  0.36779412
Sex2        -0.27613799 -0.04755236  0.18103328
Age         -0.02525772  0.02022033  0.06569837
```

#We investigated the effect of personality traits scored using the Dog Mentality Assessment (DMA) test on the latency to reach the bowl in each one of the probe locations (i.e. NP, M, NN). In the analysis below the dataset was stratified by bowl position#

Model 2.1

#Data stratification - bowl position NP#

```
> model2<-lme(log_latency~play+curiosity+chase+social+aggressive,,data=data,random=~1|Dog/MEAN,method="ML"
> summary(model2)
Linear mixed-effects model fit by maximum likelihood
 Data: data
       AIC      BIC    logLik
  120.1511 142.6494 -51.07557

Random effects:
 Formula: ~1 | Dog
        (Intercept)
StdDev:   0.1794029

 Formula: ~1 | MEAN %in% Dog
        (Intercept)  Residual
StdDev:   0.1794027 0.3682445

Fixed effects: log_latency ~ play + curiosity + chase + social + aggressive
                 Value  Std.Error DF   t-value p-value
(Intercept)  1.0423668 0.06334383 60 16.455695  0.0000
play        -0.0197033 0.07585902 24 -0.259736  0.7973
curiosity   -0.1170685 0.10231910 24 -1.144151  0.2638
chase       -0.1136511 0.07764658 24 -1.463697  0.1563
social      -0.0447136 0.09738754 24 -0.459131  0.6503
aggressive  -0.1336323 0.10296375 24 -1.297857  0.2067

Standardized Within-Group Residuals:
       Min         Q1        Med         Q3        Max
-1.7211487 -0.4594539 -0.1328030  0.2483418  4.7680088

Number of Observations: 90
Number of Groups:
          Dog MEAN %in% Dog
           30            30
```

#We run an ANOVA on the linear part of the model [library(car)] to calculate p-values for the fixed effects using Wald test#

```
> Anova(model2)
Analysis of Deviance Table (Type II tests)

Response: log_latency
            Chisq Df Pr(>Chisq)
play       0.0723  1     0.7880
curiosity  1.4026  1     0.2363
chase      2.2954  1     0.1298
social     0.2259  1     0.6346
aggressive 1.8048  1     0.1791
```

#We investigated if the model would improve by using a backward stepwise approach (i.e. dropping variables one at the time and comparing the AIC values for best fit). The model fit did not improve significantly so we used the above model as the final one#

#To further explore the reliability of our results we compared them with the confidence intervals of the model estimates [significant effect is considered if the CI does not include zero]#

```
> intervals(model2,which="fixed")
Approximate 95% confidence intervals

 Fixed effects:
                 lower        est.      upper
(Intercept)  0.9199567  1.04236681 1.16477695
play        -0.1709598 -0.01970332 0.13155315
curiosity   -0.3210842 -0.11706854 0.08694710
chase       -0.2684718 -0.11365110 0.04116962
social      -0.2388961 -0.04471360 0.14946892
aggressive  -0.3389333 -0.13363226 0.07166874
```

Model 2.2

#Data stratification - bowl position M#

```
model2<-lme(log_latency~play+curiosity+chase+social+aggressive,,data=data,random=~1|Dog/MEAN,method="ML")
> summary(model2)
Linear mixed-effects model fit by maximum likelihood
 Data: data
       AIC      BIC    logLik
  120.1511 142.6494 -51.07557

Random effects:
 Formula: ~1 | Dog
        (Intercept)
```

```
StdDev:   0.2537138

 Formula: ~1 | MEAN %in% Dog
        (Intercept)  Residual
StdDev: 6.747548e-05 0.3682445

Fixed effects: log_latency ~ play + curiosity + chase + social + aggressive
                 Value  Std.Error DF   t-value p-value
(Intercept)  1.0423668 0.06334383 31 16.455695  0.0000
play        -0.0197033 0.07585902 24 -0.259736  0.7973
curiosity   -0.1170685 0.10231910 24 -1.144151  0.2638
chase       -0.1136511 0.07764658 24 -1.463697  0.1563
social      -0.0447136 0.09738754 24 -0.459131  0.6503
aggressive  -0.1336323 0.10296374 24 -1.297857  0.2067

Standardized Within-Group Residuals:
       Min         Q1        Med         Q3        Max
-1.7211486 -0.4594539 -0.1328030  0.2483418  4.7680088

Number of Observations: 90
Number of Groups:
          Dog MEAN %in% Dog
           30            59

#We run an ANOVA on the linear part of the model [library(car)] to calculate p-values for the fixed effects
 using Wald test#
> Anova(model2)
Analysis of Deviance Table (Type II tests)

Response: log_latency
            Chisq Df Pr(>Chisq)
play       0.0723  1     0.7880
curiosity  1.4026  1     0.2363
chase      2.2954  1     0.1298
social     0.2259  1     0.6346
aggressive 1.8048  1     0.1791

#We investigated if the model would improve by using a backward stepwise approach (i.e. dropping variables one
at the time and comparing the AIC values for best fit). The model fit did not improve significantly so we used
the above model as the final one#

#To further explore the reliability of our results we compared them with the confidence intervals of the model
estimates [significant effect is considered if the CI does not include zero]#
> intervals(model2,which="fixed")
Approximate 95% confidence intervals

 Fixed effects:
                 lower        est.      upper
(Intercept)  0.9175568  1.04236681 1.16717678
play        -0.1709598 -0.01970332 0.13155315
curiosity   -0.3210842 -0.11706854 0.08694710
chase       -0.2684718 -0.11365110 0.04116962
social      -0.2388961 -0.04471360 0.14946891
aggressive  -0.3389333 -0.13363226 0.07166874
attr(,"label")
[1] "Fixed effects:"
```

Model 2.3

#Data stratification - bowl position NN#

```
> model2<-lme(log_latency~play+curiosity+chase+social+aggressive,,data=data,random=~1|Dog/MEAN,method="ML")
> summary(model2)
Linear mixed-effects model fit by maximum likelihood
 Data: data
       AIC      BIC    logLik
  245.4341 267.9324 -113.7171

Random effects:
 Formula: ~1 | Dog
        (Intercept)
StdDev:   0.2299388

 Formula: ~1 | MEAN %in% Dog
        (Intercept)  Residual
StdDev:   0.2299391 0.8005683

Fixed effects: log_latency ~ play + curiosity + chase + social + aggressive
                 Value Std.Error DF   t-value p-value
(Intercept)  1.7006355 0.1081455 60 15.725436  0.0000
play        -0.0237832 0.1295124 24 -0.183636  0.8558
curiosity   -0.0155499 0.1746871 24 -0.089016  0.9298
chase       -0.1482492 0.1325643 24 -1.118319  0.2745
social      -0.3474600 0.1662676 24 -2.089764  0.0474
aggressive  -0.0986757 0.1757877 24 -0.561335  0.5798
Standardized Within-Group Residuals:
       Min         Q1        Med         Q3        Max
-1.8292029 -0.5575156 -0.3371643  0.5155310  2.1386546
```

```
Number of Observations: 90
Number of Groups:
          Dog MEAN %in% Dog
           30            30
```

#We run an ANOVA on the linear part of the model [library(car)] to calculate p-values for the fixed effects using Wald test#

```
> Anova(model2)
Analysis of Deviance Table (Type II tests)

Response: log_latency
            Chisq Df Pr(>Chisq)
play       0.0361  1    0.84925
curiosity  0.0085  1    0.92659
chase      1.3400  1    0.24704
social     4.6790  1    0.03053 *
aggressive 0.3376  1    0.56122
```

#We investigated if the model would improve by using a backward stepwise approach (i.e. dropping variables one at the time and comparing the AIC values for best fit). The model fit did not improve significantly so we used the above model as the final one#

#To further explore the reliability of our results we compared them with the confidence intervals of the model estimates [significant effect is considered if the CI does not include zero]#

```
> intervals(model2,which="fixed")
Approximate 95% confidence intervals

 Fixed effects:
                 lower        est.       upper
(Intercept)  1.4916474  1.70063553  1.90962366
play        -0.2820200 -0.02378319  0.23445365
curiosity   -0.3638613 -0.01554989  0.33276150
chase       -0.4125711 -0.14824915  0.11607284
social      -0.6789835 -0.34745997 -0.01593645
aggressive  -0.4491816 -0.09867574  0.25183012
attr(,"label")
[1] "Fixed effects:"
```

#We investigated the effect of personality traits scored using the Canine Behavioural Assessment and Research Questionnaire (C-BARQ) test on the latency to reach the bowl in each one of the probe locations (i.e. NP, M, NN). In the analysis below the dataset was stratified by bowl position#

Model 3.1

#Data stratification - bowl position NP#

```
model1L<-lme(log_latency~SDA+ODA+DDA+DDF+SDF+NSF+SRP+AAS+EXC,,data=data,random=~1|Dog/MEAN,method="ML")
> summary(model1L)
Linear mixed-effects model fit by maximum likelihood
 Data: data
       AIC      BIC    logLik
  113.6306 145.6874 -43.81532

Random effects:
 Formula: ~1 | Dog
        (Intercept)
StdDev:   0.1104806

 Formula: ~1 | MEAN %in% Dog
        (Intercept)  Residual
StdDev:   0.1104807 0.3731596

Fixed effects: log_latency ~ SDA + ODA + DDA + DDF + SDF + NSF + SRP + AAS +      EXC
                 Value  Std.Error DF   t-value p-value
(Intercept)  1.3380843 0.19612587 58  6.822579  0.0000
SDA         -0.0627533 0.13215993 19 -0.474828  0.6403
ODA          0.0118357 0.29463419 19  0.040171  0.9684
DDA          0.2355532 0.08191894 19  2.875442  0.0097
DDF          0.2997915 0.08979550 19  3.338603  0.0035
SDF          0.0388491 0.10823577 19  0.358931  0.7236
NSF         -0.3283796 0.14698977 19 -2.234030  0.0377
SRP          0.3564868 0.12015848 19  2.966805  0.0079
AAS          0.0085552 0.07791091 19  0.109807  0.9137
EXC         -0.2868472 0.08906734 19 -3.220565  0.0045

Standardized Within-Group Residuals:
       Min         Q1        Med         Q3        Max
-1.6375697 -0.3911698 -0.1152167  0.2610419  4.7661148

Number of Observations: 87
Number of Groups:
          Dog MEAN %in% Dog
           29            29
```

```
#We run an ANOVA on the linear part of the model [library(car)] to calculate p-values for the fixed effects
 using Wald test#
> Anova(model1L)
Analysis of Deviance Table (Type II tests)

Response: log_latency
      Chisq Df Pr(>Chisq)
SDA  0.2547  1  0.6137554
ODA  0.0018  1  0.9659408
DDA  9.3420  1  0.0022397 **
DDF 12.5938  1  0.0003870 ***
SDF  0.1456  1  0.7028126
NSF  5.6391  1  0.0175646 *
SRP  9.9450  1  0.0016128 **
AAS  0.0136  1  0.9070823
EXC 11.7191  1  0.0006186 ***
---
Signif. codes:  0 â€˜***â€™ 0.001 â€˜**â€™ 0.01 â€˜*â€™ 0.05 â€˜.â€™ 0.1 â€˜ â€™ 1

#We investigated if the model would improve by using a backward stepwise approach (i.e. dropping variables one
at the time and comparing the AIC values for best fit). The model fit did not improve significantly so we used
the above model as the final one#

#To further explore the reliability of our results we compared them with the confidence intervals of the model
estimates [significant effect is considered if the CI does not include zero]#
> intervals(model1A,which="fixed")
Approximate 95% confidence intervals
 Fixed effects:
                  lower          est.       upper
(Intercept)  0.96874683  1.338084339  1.70742185
SDA         -0.32298468 -0.062753253  0.19747818
ODA         -0.56831799  0.011835744  0.59198948
DDA          0.07424948  0.235553163  0.39685685
DDF          0.12297837  0.299791508  0.47660465
SDF         -0.17427409  0.038849122  0.25197233
NSF         -0.61781195 -0.328379603 -0.03894726
SRP          0.11988697  0.356486781  0.59308659
AAS         -0.14485645  0.008555152  0.16196676
EXC         -0.46222650 -0.286847161 -0.11146782
attr(,"label")
[1] "Fixed effects:"
```

Model 3.2

#Data stratification - bowl position M#

```
model1A<-lme(log_latency~SDA+ODA+DDA+DDF+SDF+NSF+SRP+AAS+EXC,,data=data,random=~1|Dog/MEAN,method="ML")
> summary(model1A)
Linear mixed-effects model fit by maximum likelihood
 Data: data
       AIC      BIC    logLik
  214.0085 246.0653 -94.00425

Random effects:
 Formula: ~1 | Dog
         (Intercept)
StdDev: 5.040828e-05

 Formula: ~1 | MEAN %in% Dog
        (Intercept)  Residual
StdDev:   0.1457115 0.6980643

Fixed effects: log_latency ~ SDA + ODA + DDA + DDF + SDF + NSF + SRP + AAS +      EXC
                 Value Std.Error DF   t-value p-value
(Intercept)  1.4082542 0.3076045 30  4.578133  0.0001
SDA          0.1512487 0.2072819 19  0.729676  0.4745
ODA         -0.5052565 0.4620246 19 -1.093570  0.2878
DDA         -0.0791187 0.1285905 19 -0.615276  0.5457
DDF          0.0785410 0.1411949 19  0.556260  0.5845
SDF         -0.1118458 0.1699036 19 -0.658290  0.5182
NSF          0.2479561 0.2308887 19  1.073920  0.2963
SRP          0.2374706 0.1884210 19  1.260319  0.2228
AAS          0.1142012 0.1224450 19  0.932673  0.3627
EXC         -0.2778214 0.1399804 19 -1.984716  0.0618
Standardized Within-Group Residuals:
       Min         Q1        Med         Q3        Max
-1.0759679 -0.6574356 -0.3065898  0.1436126  3.4431503

Number of Observations: 87
Number of Groups:
          Dog MEAN %in% Dog
           29            57

#We run an ANOVA on the linear part of the model [library(car)] to calculate p-values for the fixed effects
 using Wald test#
Anova(model1A)
Analysis of Deviance Table (Type II tests)
```

```
Response: log_latency
     Chisq Df Pr(>Chisq)
SDA 0.6016  1    0.43798
ODA 1.3512  1    0.24507
DDA 0.4277  1    0.51311
DDF 0.3496  1    0.55433
SDF 0.4896  1    0.48409
NSF 1.3031  1    0.25365
SRP 1.7947  1    0.18036
AAS 0.9829  1    0.32150
EXC 4.4507  1    0.03489 *
---
```

#We investigated if the model would improve by using a backward stepwise approach (i.e. dropping variables one at the time and comparing the AIC values for best fit). The model fit did not improve significantly so we used the above model as the final one#

#To further explore the reliability of our results we compared them with the confidence intervals of the model estimates [significant effect is considered if the CI does not include zero]#

```
> intervals(model1A,which="fixed")
Approximate 95% confidence intervals
 Fixed effects:
                 lower         est.         upper
(Intercept)  0.8172479  1.40825419  1.999260443
SDA         -0.2569028  0.15124874  0.559400251
ODA         -1.4150128 -0.50525648  0.404499820
DDA         -0.3323216 -0.07911866  0.174084251
DDF         -0.1994809  0.07854104  0.356562960
SDF         -0.4463970 -0.11184584  0.222705308
NSF         -0.2066786  0.24795605  0.702590753
SRP         -0.1335424  0.23747059  0.608483611
AAS         -0.1269009  0.11420117  0.355303270
EXC         -0.5534520 -0.27782142 -0.002190883
attr(,"label")
[1] "Fixed effects:"
```

Model 3.3

#Data stratification - bowl position NN#

```
model1L<-lme(log_latency~SDA+ODA+DDA+DDF+SDF+NSF+SRP+AAS+EXC,,data=data,random=~1|Dog/MEAN,method="ML")
> summary(model1L)
Linear mixed-effects model fit by maximum likelihood
 Data: data
       AIC      BIC    logLik
  237.2113 269.2681 -105.6056

Random effects:
 Formula: ~1 | Dog
        (Intercept)
StdDev:   0.1389265

 Formula: ~1 | MEAN %in% Dog
        (Intercept)  Residual
StdDev:   0.1389262 0.7918972

Fixed effects: log_latency ~ SDA + ODA + DDA + DDF + SDF + NSF + SRP + AAS +      EXC
                 Value Std.Error DF   t-value p-value
(Intercept)  1.5747362 0.3667231 58  4.294074  0.0001
SDA         -0.1301788 0.2471173 19 -0.526790  0.6044
ODA         -1.0186852 0.5509174 19 -1.849070  0.0801
DDA          0.2279689 0.1531749 19  1.488291  0.1531
DDF          0.3345212 0.1679028 19  1.992350  0.0609
SDF         -0.2176682 0.2023831 19 -1.075526  0.2956
NSF          0.0359586 0.2748467 19  0.130832  0.8973
SRP          0.2603316 0.2246766 19  1.158695  0.2609
AAS          0.2148456 0.1456806 19  1.474772  0.1567
EXC         -0.3004250 0.1665413 19 -1.803907  0.0871

Standardized Within-Group Residuals:
       Min          Q1         Med          Q3         Max
-1.7259493 -0.6030631 -0.2939873  0.2936751  2.2820373

Number of Observations: 87
Number of Groups:
          Dog MEAN %in% Dog
           29            29
```

#We run an ANOVA on the linear part of the model [library(car)] to calculate p-values for the fixed effects using Wald test#

```
> Anova(model1L)
Analysis of Deviance Table (Type II tests)

Response: log_latency
     Chisq Df Pr(>Chisq)
SDA 0.3135  1    0.57551
ODA 3.8631  1    0.04936 *
DDA 2.5027  1    0.11365
```

```
DDF 4.4850  1    0.03419 *
SDF 1.3070  1    0.25294
NSF 0.0193  1    0.88940
SRP 1.5169  1    0.21808
AAS 2.4574  1    0.11697
EXC 3.6767  1    0.05518 .
---
```

#We investigated if the model would improve by using a backward stepwise approach (i.e. dropping variables one at the time and comparing the AIC values for best fit). The model fit did not improve significantly so we used the above model as the final one#

#To further explore the reliability of our results we compared them with the confidence intervals of the model estimates [significant effect is considered if the CI does not include zero]#

```
> intervals(model1A,which="fixed")
Approximate 95% confidence intervals
 Fixed effects:
                   lower       est.      upper
(Intercept)  0.884135831  1.5747362 2.26533652
SDA         -0.616768764 -0.1301788 0.35641113
ODA         -2.103477156 -1.0186852 0.06610683
DDA         -0.073642444  0.2279689 0.52958023
DDF          0.003909726  0.3345212 0.66513267
SDF         -0.616173567 -0.2176682 0.18083709
NSF         -0.505232248  0.0359586 0.57714946
SRP         -0.182071073  0.2603316 0.70273434
AAS         -0.072008854  0.2148456 0.50170005
EXC         -0.628355476 -0.3004250 0.02750554
attr(,"label")
[1] "Fixed effects:"
```
